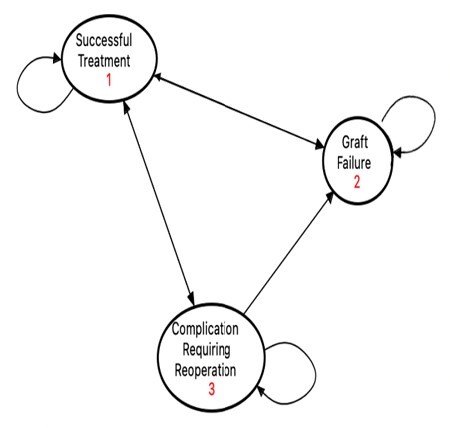# 607 Cultured Epidermal Autografts versus Spray Keratinocytes for Massive Burns—a Cost Effectiveness Analysis

**DOI:** 10.1093/jbcr/irae036.241

**Published:** 2024-04-17

**Authors:** Pooja Yesantharao, Heli Patel, Aditya Gunturi, Rahim Nazerali, Clifford C Sheckter

**Affiliations:** Stanford University, Stanford, CA; Stanford University, Menlo Park, CA; Stanford/Santa Clara Valley Medical Center, San Jose, CA; Stanford University, Stanford, CA; Stanford University, Menlo Park, CA; Stanford/Santa Clara Valley Medical Center, San Jose, CA; Stanford University, Stanford, CA; Stanford University, Menlo Park, CA; Stanford/Santa Clara Valley Medical Center, San Jose, CA; Stanford University, Stanford, CA; Stanford University, Menlo Park, CA; Stanford/Santa Clara Valley Medical Center, San Jose, CA; Stanford University, Stanford, CA; Stanford University, Menlo Park, CA; Stanford/Santa Clara Valley Medical Center, San Jose, CA

## Abstract

**Introduction:**

Massive burn injuries are challenging to treat surgically due to limited donor skin. Autologous epidermal grafts such as cultured epidermal autografts (CEAs) and spray keratinocyte suspensions are successful strategies for wound closure when donor sites are limited. There are no investigations to date that describe the differential outcomes or costs between these competing strategies. Cost-effectiveness analysis is required to guide payors, hospitals, and policy makers in determinations of care.

**Methods:**

A cost-effectiveness analysis compared CEAs with spray keratinocytes in adult burn patients with >50% total body surface area deep partial thickness burns. Hybrid Monte Carlo simulation and Markov modeling studied cost-utility from the payer perspective. Study data were derived from randomized clinical trials and cohort investigations of CEAs and spray keratinocytes. The time horizon was 1 year with 1 month cycling. Model costs included the product, surgery (both physician and facility fees), and hospitalization. Surgical costs were derived from Medicare reimbursement fee schedules. Costs of epidermal autografts were determined using currently available marketing material. All costs were inflated to 2023 US dollars, with a standard 3% discounting rate. Model utilities were derived from the Vancouver Scar Scale (VSS) with 1 as the best outcome and 0 as the worst outcome. The incremental cost-effectiveness ratio (ICER) was derived from the cost difference between the two strategies divided by the difference in adjusted VSS. Deterministic and probabilistic sensitivity analyses were performed varying all model parameters.

**Results:**

CEAs achieved successful wound closure in 73% of simulations compared to 88% for spray keratinocytes. Compared to treatment with CEAs, treatment with spray keratinocytes resulted in cost savings of $254,743, with no compromise in overall utility of treatment based on VSS. As such, spray keratinocyte treatment was the dominant strategy. This finding was robust upon sensitivity analyses.

**Conclusions:**

Spray keratinocyte suspensions were a dominant strategy to CEAs (i.e. cost saving without compromising utility).

**Applicability of Research to Practice:**

Payers and providers should consider the cost utility of spray keratinocytes as the dominant treatment strategy for massive burn epidermal grafting. CEA manufactures may want to consider cost reductions to be more economically competitive with alternative strategies.